# Three new cryptic species of *Euglossa* from Brazil (Hymenoptera, Apidae)

**DOI:** 10.3897/zookeys.222.3382

**Published:** 2012-09-21

**Authors:** André Nemésio, Michael S. Engel

**Affiliations:** 1 Instituto de Biologia, Universidade Federal de Uberlândia. Rua Ceará, s/n, Campus Umuarama, Uberlândia, MG, 38.400-902, Brazil; 2 Division of Entomology, Natural History Museum, and Department of Ecology & Evolutionary Biology, 1501 Crestline Drive – Suite 140, University of Kansas, Lawrence, Kansas 66045, USA

**Keywords:** Amazon Basin, Atlantic Forest, Apoidea, Anthophila, Euglossini, orchid bees, new species, taxonomy

## Abstract

Three new species of orchid bees are described and figured from the Amazon and Atlantic forests of Brazil. *Euglossa clausi* **sp. n.**, *Euglossa moratoi* **sp. n.**, and *Euglossa pepei* **sp. n.** are distinguished from their close congeners *Euglossa crassipunctata* Moure, *Euglossa parvula* Dressler, and *Euglossa sapphirina* Moure, previously placed in the subgenus *Euglossa* (*Glossurella*) Dressler, 1982, a demonstrably paraphyletic assemblage requiring serious reconsideration. Their affinities with related species are discussed and pertinent characters are figured.

## Introduction

The taxonomy of the Neotropical orchid bees (Apinae: Euglossini *sensu* Michener (1944, 2007) [it should be noted that the Brazilian melittological community considers this a subtribe of Apini in a less hierarchical classification of Apoidea whereby bees are relegated to a single family; the differences, however, are semantic and the concepts of included taxa are equivalent]) received a tremendous boost after the 1960s, when it was realized that males could be attracted easily to synthetic fragrances that mimic the odor of some flowers, especially orchids (Vogel 1966; Dodson et al. 1969). Many unknown species were thus captured, recognized, and subsequently described (e.g., Moure 1968, 1969, 1970; Dressler 1978, 1982a, 1982b, 1982c). Although some orchid bee species continued to be described after this flurry of activity, a period of relative taxonomic stasis developed during late 1980s and through the 1990s, until the end of the 90s when new species again began to be described (e.g., Engel 1999). In addition to a critical reappraisal of historical type material, otherwise ‘hidden’ sibling and cryptic species were recognized and this led to a new wave of descriptive work over the last decade (e.g., Oliveira and Nemésio 2003; Roubik 2004; Ramírez 2005, 2006; Parra et al. 2006; Rasmussen and Skov 2006; Nemésio 2006, 2007a, 2007b, 2008, 2009, 2010a, 2011b, 2011c, 2011d, 2012; Oliveira 2006, 2011; Bembé 2007, 2008; Hinojosa-Díaz and Engel 2007, 2011a, 2011b; Nemésio and Bembé 2008; Hinojosa-Díaz et al. 2011, 2012; Eltz et al. 2011; Faria and Melo 2011, 2012; Nemésio and Ferrari 2012).

Herein we continue this tradition with the recognition and description of three new species of *Euglossa* Latreille. All three species are closely related to species until recently placed in the paraphyletic subgenus *Glossurella* Dressler (Ramírez et al. 2010; Hinojosa-Díaz 2010, in prep.) and here left as *incertae sedis* (as suggested by Hinojosa-Díaz and Engel 2011b; Hinojosa-Díaz et al. 2012). Two of the new species, *Euglossa clausi* sp. n. and *Euglossa moratoi* sp. n., are closely related to the Central American *Euglossa crassipunctata* Moure and *Euglossa sapphirina* Moure and have been identified as *Euglossa crassipunctata* both in the Amazon and Atlantic forests. However, the species can be differentiated not only on the basis of coloration and size, but also in the male terminalia. The third species, *Euglossa pepei* sp. n., is described from the Atlantic forest of southern Bahia, and is one of the most distinctive, apparently sharing some characters with the Amazonian *Euglossa parvula* Dressler, but differing in terms of its genitalia.

## Material and methods

Material considered herein is deposited in the collections of the Universidade Federal de Minas Gerais, Belo Horizonte, Brazil (**UFMG**); Florida Museum of Natural History, Gainesville, Florida, USA (**FMNH**); and the Division of Entomology, University of Kansas Natural History Museum, Lawrence, Kansas, USA (**SEMC**). General morphological terminology for bees follows Engel (2001) and Michener (2007), while specific terms for orchid bees follows Engel (1999), Nemésio (2009: 10, 12), and Hinojosa-Díaz (2008). Metasomal terga and sterna are referred to as T1, T2, ... T*n*, and S1, S2, ... S*n*, respectively. Integumental and setal coloration are those observed by eye under a Leica MZ12 or Olympus SZX-12 stereomicroscope with reflected fiber optic illumination. Measurements provided are those of the name-bearing holotypes. The taxonomic arrangement of genera, subgenera, and species adopted herein follows that of Nemésio and Rasmussen (2011). Material of representative other euglossine species was examined from UFMG, SEMC, and FMNH. Label data are given with each label separated by “”. When data of a label of the subsequent specimen are identical to those of the previously cited specimen, only “idem” is provided. We have provided genitalic characters to distinguish the species. There is variation in the genitalia, particularly in the form of the gonostylus, within some species of *Euglossa* but the presence of such variation within an individual species is not consistent throughout the genus (e.g., Hinojosa-Díaz and Engel 2011a). For the moment there does not appear to be significant genitalic variation within the species considered herein but this should be clarified should the new species herein be discovered at more distant geographic locales. Regardless of this variation, important characters for the recognition of species, species groups, and even larger clades are present within Euglossini (Hinojosa-Díaz 2008).

## Systematics

### Genus *Euglossa* Latreille

All three species described herein are left as *incertae sedis* in regard to subgenus (following the suggestion of Hinojosa-Díaz and Engel 2011b; Hinojosa-Díaz et al. 2012), but they share a number of characters which suggest they are closely related such as: small bees with dark blue clypeus, very coarsely punctate mesepisternum and coarsely punctate mesoscutum, anterior mesotibial tuft entire, sternal tufts in semi-circular depressions. Specific characters of each species are given below, as well as between the new species and *Euglossa crassipunctata* and *Euglossa sapphirina* in the diagnoses and discussion (*vide infra*).

#### Euglossa clausi sp. n.

urn:lsid:zoobank.org:act:D52A8C97-75AD-4F25-8450-B0CC4DA5D103

http://species-id.net/wiki/Euglossa_clausi

**Figures 1–3.**
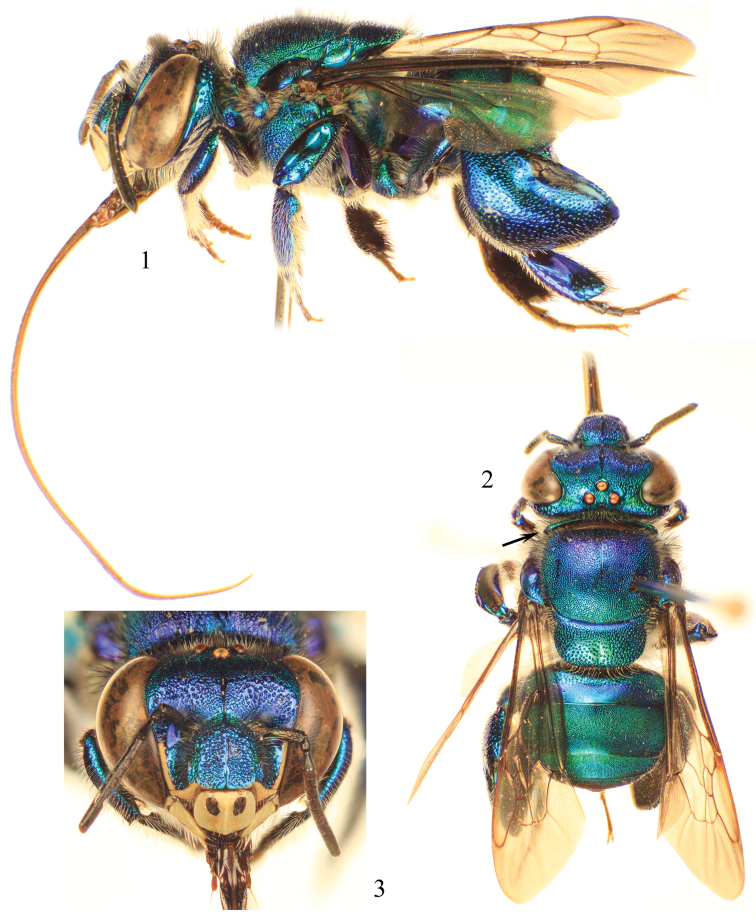
Photomicrographs of paratype male of *Euglossa clausi* Nemésio and Engel, sp. n. **1** Lateral habitus **2** Dorsal habitus (arrow points to rounded pronotal angle) **3** Facial aspect.

Figures 1 [Fig F2] 11

##### Holotype.

♂, with the following data: “Euglossini do PERD, Pq. E. Rio Doce, 3859-11105” and “Marliéria, MG, Brasil, 04/07/1999, A. Nemésio” (UFMG). Details of the type locality are: Parque Estadual do Rio Doce (19°43'S, 42°34'W; 200 m a.s.l.), in the municipality of Marliéria, state of Minas Gerais, southeastern Brazil.

##### Paratypes.

3♂♂, with the following label data: “Euglossini do PERD, Pq. E. Rio Doce, 3859-11106” and “Marliéria, MG, Brasil, 04/07/1999, A. Nemésio”; “idem, 3872-11131” and “idem” (UFMG); “idem, 3876-11137” and “idem” (UFMG). 1♂, “Brazil, E. Santo, No. Linhares, 12.xi.1968, R.L. Dressler” (FMNH). 1♂, “Brazil, Bahia, Res. Mte. Pascoal, 8.xi.1968, R.L. Dressler” (FMNH). 1♂, “Brazil, E. Santo, Conceicao da Barra, 10.xi.1968, R.L. Dressler” (FMNH). 1♂, “Brazil, Bahia, Itabuna, 19.vi.1971, H. Kennedy, cineole” (SEMC). 1♂, “Brazil, Bahia, Itabuna, 6.xi.1968, R.L. Dressler” (SEMC).

##### Diagnosis.

*Euglossa clausi* can be distinguished readily from both *Euglossa crassipunctata* and *Euglossa sapphirina* owing to its larger size (ca. 15% larger than both species), and a combination of integumental coloration that exactly matches neither of the aforementioned species (and for this reason has been confused with both: *vide* Nemésio 2009: 85–87). The paraocular ivory markings in *Euglossa clausi* are wider below (Fig. 3) than in both *Euglossa crassipunctata* and *Euglossa sapphirina*. The metatibia and sterna (Figs 1, 6) are blue, contrasting the otherwise green metasoma, a color combination not found in *Euglossa crassipunctata* (green metasoma, including the sterna, and metatibia) and *Euglossa sapphirina* (blue throughout). The apical setae of S7 of *Euglossa clausi* are distributed throughout the invaginated section and the posterolateral projections of the anterior section of S8 angled but not prominent, instead being more strongly developed in *Euglossa moratoi* (Figs 7, 8), as is the development of the basolateral projections of the posterior section. The gonostylus of *Euglossa clausi* is more straight or even slightly downcurved (Figs 9–11), relative to that of *Euglossa moratoi* (Figs 23–25), and both differ from the terminalia of *Euglossa crassipunctata* (Figs 12–15).

##### Description.

♂: Body length ca. 10.0 mm; forewing length ca. 7.7 mm; head width 4.4 mm; interorbital distance at level of antennal sockets 2.5 mm; maximum interorbital distance 2.7 mm; labiomaxillary complex in repose reaching tip of body; scape length 0.8 mm; compound eye length 2.7 mm; mesoscutellum width 2.5 mm, length 1.2 mm; abdominal width 4.2 mm.

**Figures 4–6. F2:**
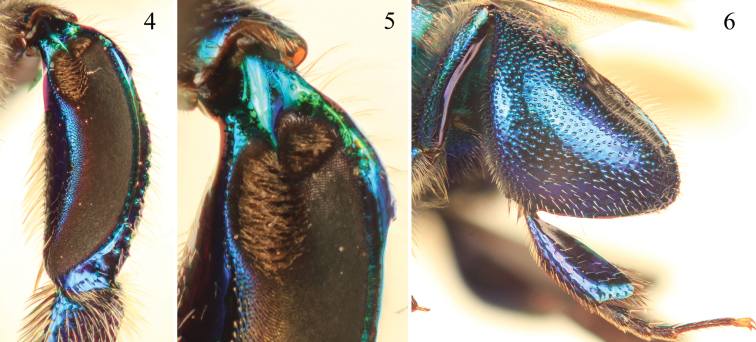
Tibial characters of *Euglossa clausi* Nemésio and Engel, sp. n. **4** Outer surface of mesotibia **5** Detail of mesotibial tufts **6** Outer surface of metatibia.

*Coloration and vestiture*: Clypeus and upper frons dark blue, remainder of head greenish-blue (Fig. 3); ivory paraocular markings well developed, reaching malar area, wider below; anterior surface of antennal scape black with very minute ivory marking in some specimens (including holotype); mesoscutum, mesoscutellum, and metasoma bluish-green (Figs 1, 2). Wing membranes lightly infumate. Pubescence very sparse, predominantly fulvous setae on metasoma and around antennal sockets, black and fulvous setae on mesosoma, black setae especially on mesoscutum (compared to predominantly fulvous setae in *Euglossa moratoi*). Protibia and probasitarsus fringed with dense fulvous setae; velvet area occupying all ventral surface of mesotibia, posterior mesotibial tuft approximately one-third size of anterior tuft, almost an isosceles triangle in shape, merging with anterior tuft; anterior mesotibial tuft oval, about three times larger than posterior tuft (Figs 4, 5); metatibia oblong-rhomboid, inflated (Fig. 6).

*Punctation*: Mesoscutum with punctation separated by a puncture width or less, with large circular punctures; punctures on mesoscutellum sparser than on mesoscutum medioposteriorly, separated there by a puncture width or greater, with larger circular punctures. Punctation on discal base of T1 with large circular punctures of roughly same size more clearly defined medially than in other species and separated by less than a puncture width; punctures of T1–T6 dense, comprised of minute circular punctures; punctures on T7 sparser than on preceding terga, with large circular punctures; S2 with small, widely-separated tufts.

**Figures 7–11.**
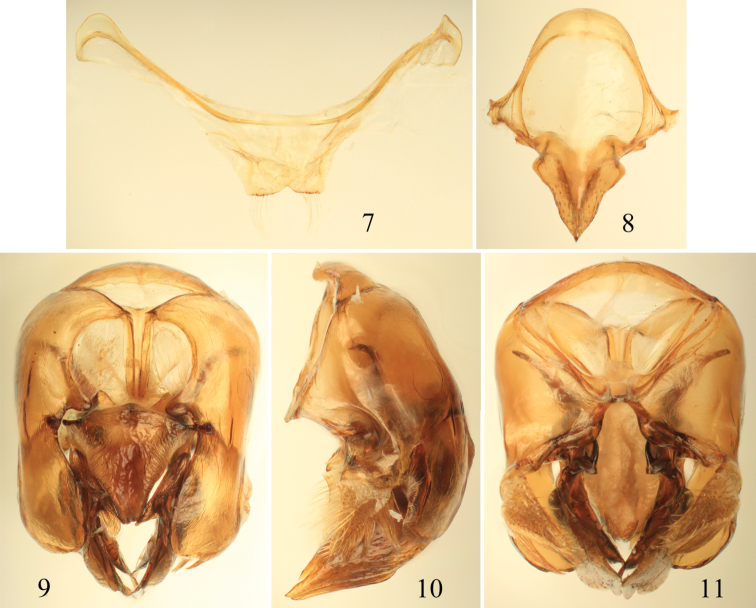
Male terminalia of *Euglossa clausi* Nemésio and Engel, sp. n. **7** Seventh metasomal sternum **8** Eighth sternum (note that relative proportions of the anterior section to the posterior section may be distorted owing to position of sclerite when photographed) **9** Genital capsule, dorsal view **10** Genital capsule, lateral view **11** Genital capsule, ventral view.

*Terminalia*: Male terminalia as in figures 7–11. S7 slightly invaginated mesally, forming a shallow incision with converging sides forming angle of ~110°, lateral sections faintly curved; apical setae throughout invaginated section, comprising seven alveoli (with one seta each) on each side; notospiculum weak, slightly divided apically, posterolateral projections of anterior section weak, not prominent; posterior section triangular, sharply pointed, with basolateral points not as sharply developed as in *Euglossa moratoi*, slightly more rounded; anterior-most section of gonobase projected ventrally, forming angle of ~100° with remainder of ventral edge; gonostylus simple (‘type V’ of Ospina-Torres et al. 2006), lateral lobe pointed and slightly curved downwards; gonostylar setae long throughout; dorsal process of gonocoxa well developed, apical process evenly rounded laterally.

♀: Unknown.

##### Etymology.

The specific epithet is a patronym honoring Dr. Claus Rasmussen, noted corbiculate bee biologist and systematist, in recognition of his years of kind collegiality.

##### Baits.

Specimens of this species have been collected mostly from baits of cineole and vanillin, while a few specimens were collected from skatole.

##### Geographic distribution.

*Euglossa clausi* sp. n. is a widespread bee in the Atlantic forest. Males have been collected from the state of Pernambuco in the north, to the northern portion of the state of São Paulo in the south (*vide* Nemésio 2009: 115 for specific locations where this species has been recorded).

##### Comments.

Specimens of this species had been labeled in collections under the nomen nudum “*cyanifrons*”. It may be that additional material is located in other institutions under this name. In addition, individuals of this species were treated in the literature as *Euglossa sapphirina* (Tonhasca et al. 2002a, 2002b, 2003; Neves and Viana 2003; Nemésio and Silveira 2006, 2007) or *Euglossa crassipunctata* (Milet-Pinheiro and Schlindwein 2005; Moura and Schlindwein 2009; Nemésio 2009, 2010b, 2011a, 2011b).

#### Euglossa moratoi sp. n.

urn:lsid:zoobank.org:act:78CE101D-7A27-47AD-8C6D-A980E636A432

http://species-id.net/wiki/Euglossa_moratoi

Figures 16 [Fig F6] 25

##### Holotype.

♂, with the following data: “EIA Porto Trombetas, Cipó I, Zona Leste, 12200-36025” and “Oriximiná, PA, Brasil 25/02/2007, R. B. Martines” (UFMG). The type locality is: Porto Trombetas, in the municipality of Oriximiná, state of Pará, northern Brazil.

##### Paratypes.

10 ♂♂, with the following label data: “EIA Porto Trombetas, Monte Branco 2, Zona Leste, 11567-34328” and “Oriximiná, PA, Brasil 11/12/2006, R. B. Martines” (UFMG); “idem, 11575-34366” and “idem” (UFMG); “idem, 11578-34374” and “idem” (UFMG); “idem, Cipó 2, Zona Leste, 11634-34512” (SEMC) and “idem, 13/12/2006” and “idem” (UFMG); “idem, Teófilo 2, Zona Leste, 11545-34254” and “idem, 10/12/2006” (UFMG); “ParNa S. do Divisor, 12512-36708” and “Mâncio Lima, AC, Brasil, 21/11/1996, E. F. Morato” (UFMG); “idem, 12541-36759” and “idem” (UFMG); “14507-42692” and “Santarém, PA, Brasil, 11/12/1978, A. Raw”, (UFMG); “14917-43369” and “Manaus, AM, Brasil, 08/10/1988, E. F. Morato” (UFMG); “Santa Maria, 04°13'S, 55°58'W, 14396-42535” and “Itaituba, PA, Brasil, 18/01/1979, J. M. F. Camargo” (UFMG).

##### Diagnosis.

*Euglossa moratoi* sp. n. can be distinguished most easily from *Euglossa crassipunctata*, *Euglossa sapphirina*,and *Euglossa clausi* due to its small size (ca.20% smaller than the other species), the projecting pronotal dorsolateral angle which is more acute (slightly pointing) at its apex (differing from the rather bluntly rounded and non-projecting angle in all other species in the *crassipunctata* group) (Fig. 17; *cf*. figure 2), and the longer posterior mesotibial tuft relative to those in *Euglossa crassipunctata*, *Euglossa sapphirina*, and *Euglossa clausi* (Figs 19, 20); photographs of the holotypes of *Euglossa crassipunctata* and *Euglossa sapphirina* are in Nemésio 2009: 87). The paraocular ivory markings in *Euglossa moratoi* are not as wide below as in the other three species (Fig. 18). Moreover, *Euglossa moratoi* is the least bluish of all four species in this complex, with bluish coloration only on the clypeus and upper frons, mesoscutum, and S2 (Figs 16–18), although there is some variation whereby the blue is slightly more extensive but still always less so than the other species. *Euglossa crassipunctata* and *Euglossa clausi*, on the other hand, have strong bluish hues on the metasoma, particularly the sterna and also on the metatibia in the latter species. *Euglossa sapphirina* is an entirely bluish-violet bee. The apical setae of S7 of *Euglossa moratoi* are restricted to the very outer sides of the invaginated section, whereas such setae are distributed throughout the invaginated section in *Euglossa clausi*, although these sterna are otherwise virtually identical between the two species. The posterolateral projections of the anterior section of S8 in *Euglossa moratoi* are strongly prominent and angled (Fig. 22), while they are distinctly weaker in *Euglossa clausi*, as is the development of the basolateral projections of the posterior section. The gonostylus of *Euglossa moratoi* is comparatively shorter than in *Euglossa clausi* and slightly upcurved (in *Euglossa clausi* it is more straight or even slightly downcurved) (Figs 23–25). *Euglossa moratoi* is among the smallest of all *Euglossa*. While the holotype is approximately 8.0 mm in length, some specimens barely exceed 7.0 mm.

##### Description.

♂: Body length ca. 8.0 mm; forewing length ca. 6.7 mm; head width 3.7 mm; interorbital distance at level of antennal sockets 2.1 mm; maximum interorbital distance 2.2 mm; labiomaxillary complex in repose reaching tip of body; scape length 0.56 mm; compound eye length 2.4 mm; mesoscutellum width 2.0 mm, length 0.93 mm; abdominal width 3.4 mm.

*Coloration and vestiture*: Clypeus and upper frons dark blue, remainder of face greenish (Fig. 18); ivory paraocular markings well developed, reaching malar area, not very wide below; anterior surface of antennal scape black; mesoscutum bluish-green, mesoscutellum and metasoma green (Figs 16, 17). Wing membranes lightly infumate. Pubescence very sparse, predominantly fulvous on metasoma and around antennal sockets, black and fulvous setae on mesosoma (compared to predominantly black setae in *Euglossa clausi*). Protibia and probasitarsus fringed with dense, fulvous setae; velvet area occupying all ventral surface of mesotibia, posterior mesotibial tuft approximately nearly one-third size of anterior tuft, triangular, slightly long and merging with anterior tuft; anterior mesotibial tuft oval, 2.5 times larger than posterior tuft (Figs 19, 20); metatibia oblong-rhomboid, inflated (Fig. 21).

*Punctation*: Mesoscutum with large circular punctures separated by a puncture width or less except anteromedially separated by a puncture width or greater particularly medially; punctures on mesoscutellum sparser than on disc of mesoscutum, with larger circular punctures separated by a puncture width or greater except along borders punctures separated by less than a puncture width. Punctation on discal base of T1 with large circular punctures of roughly same size more clearly defined medially and separated by less than a puncture width; punctation on T1–T6 dense, comprised of small hexagonal punctures; on T7 sparse relative to preceding terga, with large circular punctures; S2 with very small, widely-separated, semicircular tufts.

*Terminalia*: Male terminalia as in figures 22–25. S7 largely as in *Euglossa clausi*, with posterior margin of S7 slightly invaginated mesally, forming a shallow incision with converging sides forming an angle of ~110°, lateral sections slightly curved; apical setae only on outer sides of invaginated section, comprising four alveoli (with one seta each) on each side; notospiculum weak, slightly divided apically, posterolateral projections of anterior section large and pronounced; posterior section triangular, sharply pointed apically, with prominent basolateral points; anteriormost section of gonobase curved ventrally forming an angle of ~100° with remainder of ventral edge; gonostylus simple (‘type V’ of Ospina-Torres et al. 2006), lateral section with lobe pointed and slightly curved upwards (*sensu* Hinojosa-Díaz 2008); gonostylar setae long throughout; dorsal process of gonocoxa well developed, apical process evenly rounded laterally.

♀: Unknown.

##### Etymology.

The specific epithet is a patronym honoring Dr. Élder Ferreira Morato, noted entomologist and close colleague of the senior author.

##### Baits.

Specimens of this species have been collected mostly at baits of vanillin, although a few specimens were also attracted to cineole, eugenol, and skatole.

##### Geographic distribution.

*Euglossa moratoi* seems to be widespread in the Amazon Basin. Males have been collected from the westernmost part of the Brazilian Amazon (Nemésio and Morato 2004, 2006; Storck-Tonon et al. 2009; Oliveira et al. 2010) to the state of Pará in the east, where the holotype and some paratypes were collected. We have not examined the individuals identified as *Euglossa crassipunctata* in Rasmussen (2009), but it is possible that those also belong to *Euglossa moratoi* or perhaps yet another undescribed species (this seems the most likely of the two scenarios).

##### Comments.

Specimens of this species have been treated as *Euglossa crassipunctata* in the literature (Nemésio and Morato 2004, 2006; Storck-Tonon et al. 2009; Oliveira et al. 2010).

**Figures 12–15.**
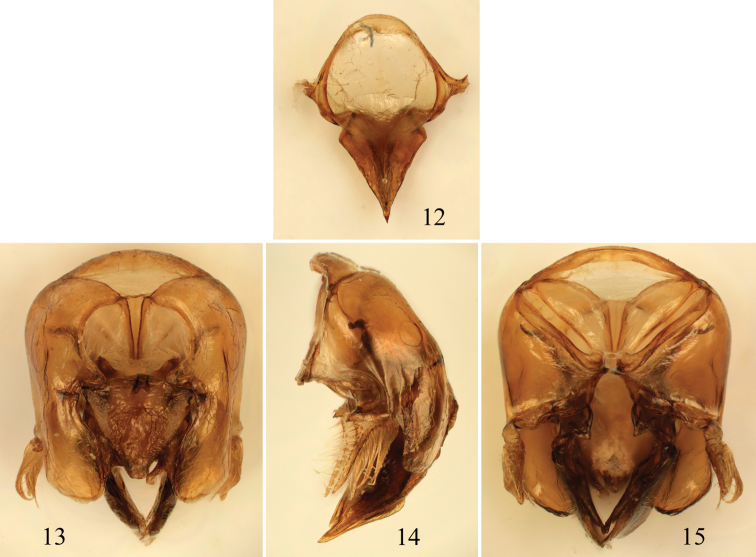
Male terminalia of *Euglossa crassipunctata* Moure. **12** Eighth metasomal sternum (note that relative proportions of the anterior section to the posterior section may be distorted owing to position of sclerite when photographed) **13** Genital capsule, dorsal view **14** Genital capsule, lateral view **15**  Genital capsule, ventral view.

**Figures 16–18.**
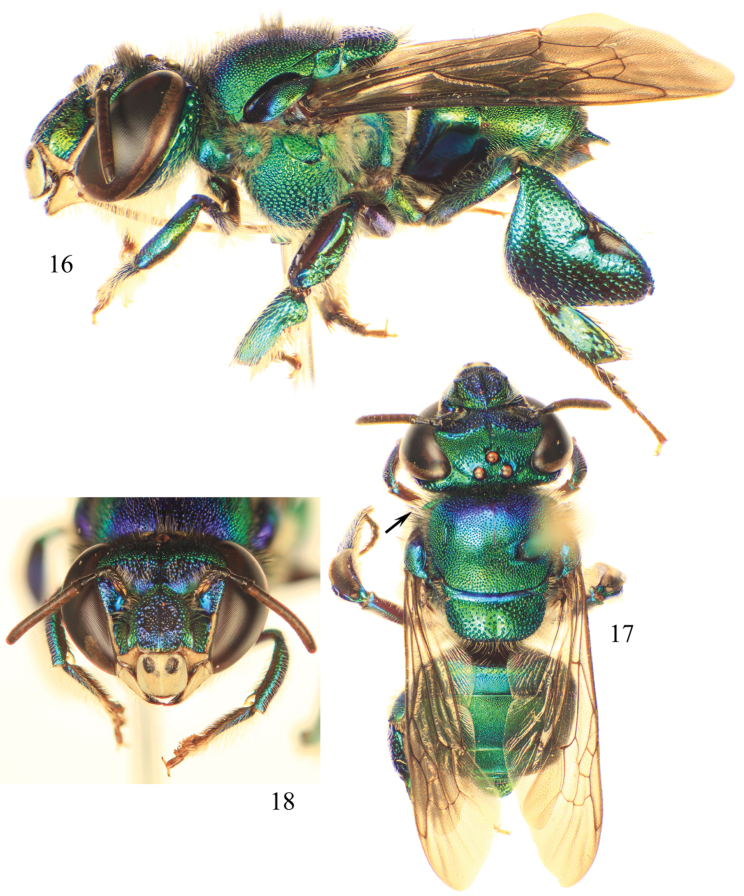
Photomicrographs of paratype male of *Euglossa moratoi* Nemésio and Engel, sp. n. **16** Lateral habitus **17** Dorsal habitus (arrow points to projected pronotal angle) **18** Facial aspect.

**Figures 19–21. F6:**
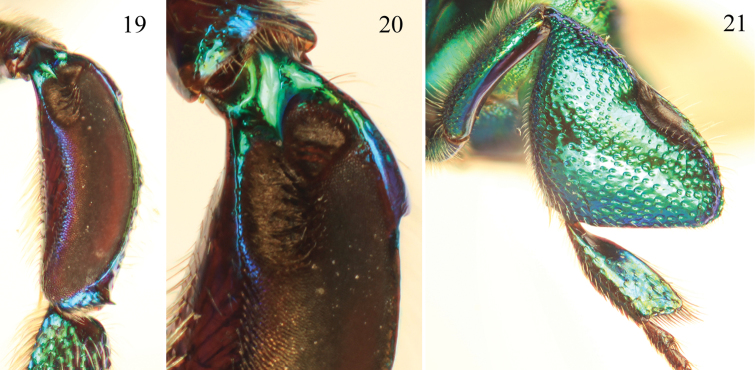
Tibial characters of *Euglossa moratoi* Nemésio and Engel, sp. n. **19** Outer surface of mesotibia **20** Detail of mesotibial tufts **21** Outer surface of metatibia.

#### Euglossa pepei sp. n.

urn:lsid:zoobank.org:act:FFEAFD21-E49B-4313-9A5D-27313107095B

http://species-id.net/wiki/Euglossa_pepei

Figures 26 [Fig F9] 36

##### Holotype.

♂, with the following data: “Euglossina da Hileia Baiana, PN Pau Brasil, 19679-56729” and “Porto Seguro, BA, Brasil, 19/04/2009, A. Nemésio” (UFMG). Details of the type locality are: Parque Nacional do Pau Brasil (16°31'S, 39°17'W; 90 m a.s.l.), in the municipality of Porto Seguro, state of Bahia, northeastern Brazil.

##### Paratypes.

4♂♂, with the following label data: “Euglossina da Hileia Baiana, PN Pau Brasil, 19641-56644” and “Porto Seguro, BA, Brasil, 17/04/2009, A. Nemésio” (UFMG); “idem, 19659-56671” and “idem, 18/04/2009” (UFMG); “idem, 19706-56790” and “idem, 20/04/2009” (UFMG), and “Euglossina da Hileia Baiana, PN Descobrimento, 20601-58992” and “Prado, BA, Brasil, 18/12/2008, A. Nemésio” (SEMC).

##### Diagnosis.

*Euglossa pepei* is the most distinctive of the species of *crassipunctata* group. The shape and size of the anterior mesotibial tuft and the presence of a minute posterior tuft (Figs 29, 30) is most similar to that observed in *Euglossa parvula* Dressler. However, both species can be separated by the larger size of the oval anterior tuft and the smaller glandular scar of the metatibia in *Euglossa pepei*. In regards to the terminalia, S8 in *Euglossa pepei* is distinctly more slender (*cf*. figures 33 and 37), and the gonostylus is more pronounced (*cf*. figures 34–36 versus 38–40). In addition, the bluish coloration is practically restricted to the head and discal base of the mesoscutum, and the sterna are golden green, the latter feature contrasting with other species in the group for which there are at least present some bluish hues.

##### Description.

♂: Body length ca. 9.5 mm; forewing length ca. 7.7 mm; head width 3.7 mm; interorbital distance at level of antennal socket 2.1 mm; maximum interorbital distance 2.6 mm; labiomaxillary complex in repose reaching apex of body; scape length 0.7 mm; compound eye length 2.7 mm; mesoscutellum width 2.3 mm, length 1.1 mm; abdominal width 3.8 mm.

*Coloration and vestiture*: Clypeus and upper frons dark blue, remainder of head greenish (Fig. 28); ivory paraocular markings well developed, reaching malar area but not particularly wide below; anterior surface of antennal scape black; discal base of mesoscutum blue, remainder of mesoscutum, mesoscutellum, and metasoma green (Figs 26, 27). Wing membranes lightly infumate. Pubescence very sparse, predominantly fulvous setae on metasoma and around antennal sockets, black and fulvous setae on mesosoma, black setae especially prominent on mesoscutum (compared to predominantly fulvous setae in *Euglossa parvula*). Protibia and probasitarsus fringed with dense, fulvous setae; velvet area occupying all ventral surface of mesotibia, posterior mesotibial tuft very small, less than 1/30 of area of anterior tuft; anterior mesotibial tuft oval, very large, occupying approximately one quarter of velvet area length (Figs 29, 30); metatibia oblong-rhomboid, inflated (Fig. 31).

*Punctation*: Mesoscutum with circular punctures of two different sizes separated by less than a puncture width, those anterolaterally nearly contiguous; punctures on mesoscutellum more widely spaced than those of mesoscutal disc, with larger circular punctures separated by a puncture width or slightly less in medial third otherwise separated by less than a puncture width. Punctation on discal base of T1 with large circular punctures, punctures weak and separated by less than a puncture width; on distal part of T1 and T2–T6 dense, consisting of minute circular punctures; on T7 dense, with large circular punctures; S2 with very small, almost inconspicuous, widely-separated tufts.

*Terminalia*: Male terminalia as in figures 32–36. Posterior margin of S7 deeply invaginated mesally, lateral sections almost straight; apical setae only on two apexes of invaginated section; notospiculum weak, slightly divided apically, posterolateral projects distinct (in this regard more similar to *Euglossa clausi*, *Euglossa moratoi*, and *Euglossa parvula*); posterior section triangular, elongate, pointed apically, with basolateral projections not as prominent as in *Euglossa clausi* and *Euglossa moratoi*; anteriormost section of gonobase curved ventrally, forming angle of ~110° with remainder of ventral edge; gonostylus simple (‘type V’ of Ospina-Torres et al. 2006), lateral lobe long, pointed and almost straight; gonostylar setae short throughout; dorsal process of gonocoxa well developed, apical process evenly rounded laterally (less regularly rounded in *Euglossa parvula*).

♀: Unknown.

##### Etymology.

The specific epithet is a patronym honoring Leandro Mattos Santos, nicknamed “Pepê”, in recognition of his accomplishments in melittology.

##### Baits.

All four of the known males were collected at baits of vanillin.

##### Geographic distribution.

*Euglossa pepei* is known only from the small type series, all collected at Parque Nacional do Pau Brasil, municipality of Porto Seguro, Bahia, Brazil.

**Figures 22–25.**
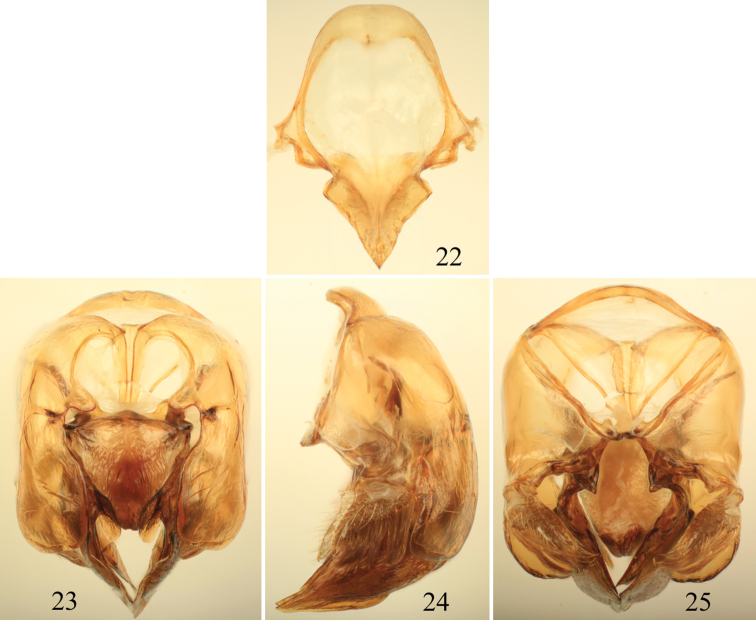
Male terminalia of *Euglossa moratoi* Nemésio and Engel, sp. n. **22** Eighth metasomal sternum (note that relative proportions of the anterior section to the posterior section may be distorted owing to position of sclerite when photographed) **23** Genital capsule, dorsal view **24** Genital capsule, lateral view **25** Genital capsule, ventral view.

**Figures 26–28.**
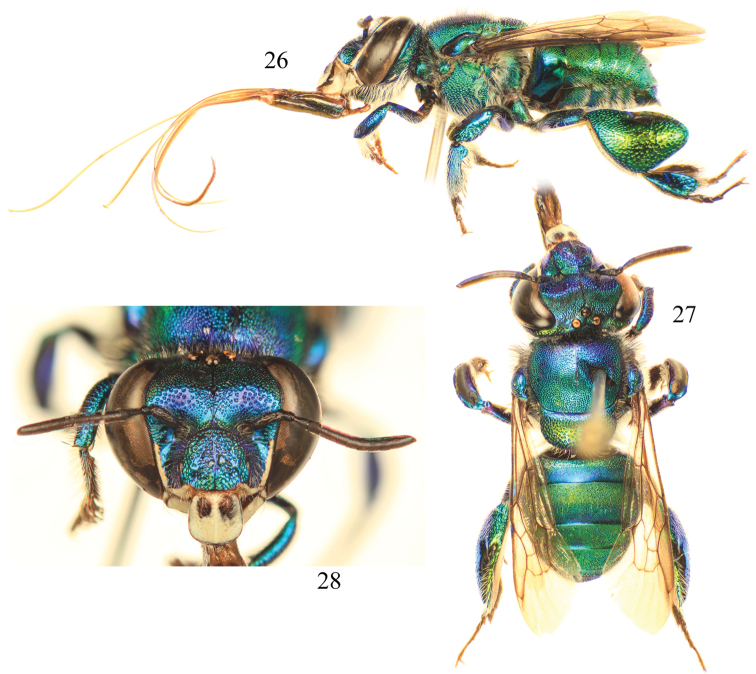
Photomicrographs of paratype male of *Euglossa pepei* Nemésio and Engel, sp. n. **26** Lateral habitus **27** Dorsal habitus **28** Facial aspect.

**Figures 29–31. F9:**
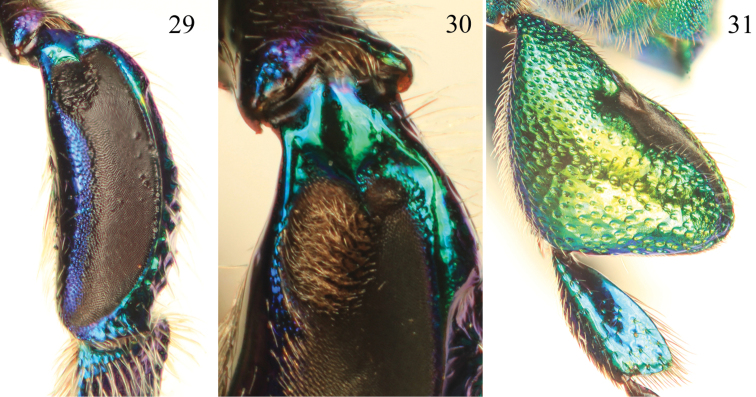
Tibial characters of *Euglossa pepei* Nemésio and Engel, sp. n. **29** Outer surface of mesotibia **30** Detail of mesotibial tufts **31** Outer surface of metatibia.

**Figures 32–36.**
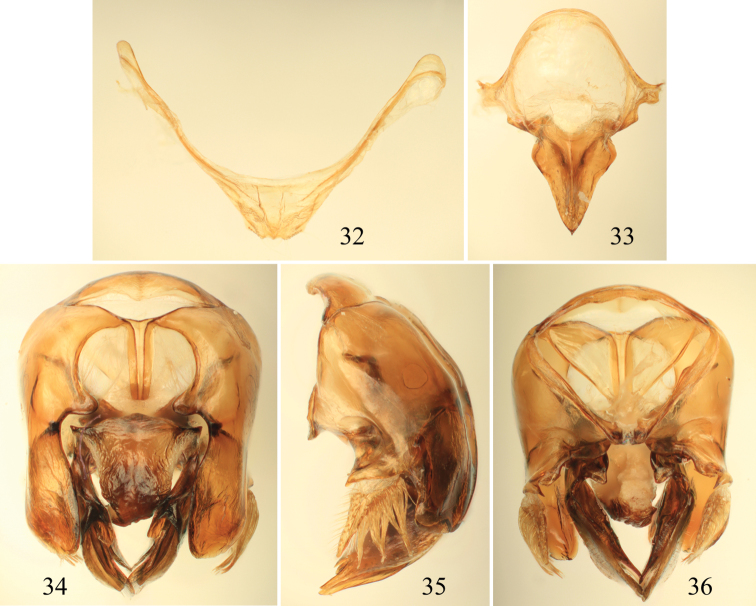
Male terminalia of *Euglossa pepei* Nemésio and Engel, sp. n. **32** Seventh metasomal sternum **33** Eighth sternum (note that relative proportions of the anterior section to the posterior section may be distorted owing to position of sclerite when photographed) **34** Genital capsule, dorsal view **35**  Genital capsule, lateral view **36** Genital capsule, ventral view.

**Figures 37–40.**
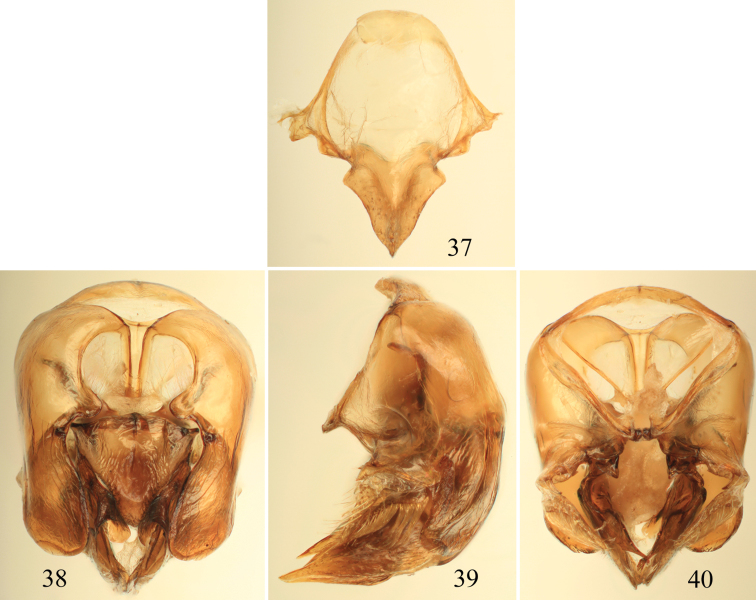
Male terminalia of *Euglossa parvula* Dressler. **37** Eighth metasomal sternum (note that relative proportions of the anterior section to the posterior section may be distorted owing to position of sclerite when photographed) **38** Genital capsule, dorsal view **39** Genital capsule, lateral view **40** Genital capsule, ventral view.

### Key to species of the *crassipunctata* group

The following key is based on males given that females are not yet known for all of the included species.

**Table:** 

1	Posterior mesotibial tuft at most small and inconspicuous, at most as wide as bordering posterior area of depressed integument (e.g., Figs 29, 30)	2
–	Posterior mesotibial tuft well developed and triangular, much larger and encompassing nearly entire bordering area of depressed integument (e.g., Figs 4, 5, 19, 20)	3
2	Mesoscutellum with punctures medially separated by a puncture width or less (rarely more so); male terminalia as in figures 32–36	*Euglossa pepei* sp. n.
–	Mesoscutellum with punctures medially a puncture width or frequently more; male terminalia as in figures 37–40	*Euglossa parvula* Dressler, 1982
3	S8 with posterolateral projections of anterior section prominently angled (e.g., Fig. 12)	4
–	S8 posterolateral projections of anterior section not developed, rounded (e.g., Figs 8, 22)	5
4	Pronotal dorsolateral angle rounded, not projecting (Fig. 2); mesoscutellum with punctures of most of disc separated by a puncture width or less except medioposteriorly some wider than a puncture width; posterolateral projection of anterior section of S8 angled but not prominent (Fig. 8)	*Euglossa clausi* sp. n.
–	Pronotal dorsolateral angle projecting, acute (Fig. 17); mesoscutellum with punctures of most of disc separated by a puncture width or slightly more and distinctly more medioposteriorly; posterolateral projection of anterior section of S8 angled and strongly prominent (Fig. 22)	*Euglossa moratoi* sp. n.
5	Mesoscutum, mesoscutellum, and majority of mesoscutum brilliant metallic green; S8 apically coming to a sharp, narrow point; gonostylus with broader base	*Euglossa crassipunctata* Moure, 1968
–	Integument entirely dark metallic blue to bluish violet; S8 apically coming to a broad point; gonostylus with narrow base	*Euglossa sapphirina* Moure, 1968

## Discussion

Recent phylogenetic studies on the interrelationships among species of *Euglossa* and based on both morphological and DNA sequence data (Ramírez et al. 2010; Hinojosa-Díaz 2010, in prep.), have highlighted that many of the traditionally recognized groups, either subgenera or species assemblages, are likely monophyletic. Nonetheless, these works have also highlighted those few groups whose monophyly remains suspect and are in need of careful attention. Most notably among those are the subgenera *Glossura* Cockerell and *Glossurella*. In regard to the former, Nemésio and Ferrari (2011) suggested that the simple synonymy of *Glossuropoda* Moure under *Glossura* would rectify the difficulty. The situation of *Glossurella* is more problematic and certainly more detailed phylogenetic studies and, perhaps most critically, the redescription of historical material and documentation of additional species within this assemblage would perhaps most greatly illuminate possible solutions. Documenting further species, such as the three described herein, enhances our understanding of variation and diversity within *Glossurella* and provides further taxa for use in future more comprehensive phylogenetic studies of the group.

When establishing *Glossurella*, Dressler (1982b) suggested the subdivision of the subgenus into species groups, the first of those comprising *Euglossa crassipunctata*, *Euglossa sapphirina*, and *Euglossa parvula*. All three species are quite similar superficially and are also among the smallest of orchid bees. Moure (1968) particularly emphasized the presence of dense punctation, with small punctures on the sixth metasomal tergum, a character which, according to him, was only present in *Euglossa crassipunctata* and *Euglossa sapphirina*. The three species described herein, as well as *Euglossa parvula* (unknown to Moure in 1968), also share this particular character, reinforcing their mutual affinity.

After describing *Euglossa crassipunctata* and *Euglossa sapphirina*, Moure (1968: 43) mentioned that he was unable to find morphological features distinguishing both species outside of their coloration, preferring to erect the two taxa given that he could not find intermediates. The possibility of polymorphic species occurred to Moure (1968) and later to Nemésio (2009), who argued that,

“... in favor of this hypothesis is the fact that both species are morphologically indistinguishable, except for coloration... and that they occur sympatrically – at least *Eg. crassipunctata* is sympatric with *Eg. sapphirina* in the entire distributional range of the latter. Against this hypothesis is the fact that, strangely, the possible polymorphism is restricted to a relatively small area of the wide geographic range of *Eg. crassipunctata*.” (Nemésio 2009: 86).

At the time the above statements were made the populations considered herein as two distinct species, *Euglossa moratoi* and *Euglossa clausi*, were treated as *Euglossa crassipunctata*. Our revised interpretation of this material restricts the geographic distribution of *Euglossa crassipunctata* to Central America, where it is sympatric with *Euglossa sapphirina*. More importantly, there are slight differences in the structure of the male terminalia of both species, particularly in the form of S8 between Central American populations (MSE pers. obs.). As noted above, variation within a species for some genitalic structures is known (e.g., Hinojosa-Díaz and Engel 2011a) but this is not consistent across the genus and for many they can be relatively fixed. Molecular data may be of aid in clarifying the status of these two taxa.

While *Euglossa clausi* and *Euglossa moratoi* are remarkably similar superficially to *Euglossa crassipunctata*, the form of the male terminalia serves to most strongly distinguish these species. For instance, the posterolateral projection of the anterior section of S8 in *Euglossa crassipunctata* is scarcely developed and gently rounded, while this process if more developed in the new species, each forming a noticeable angle, although it is most extremely developed in *Euglossa moratoi*. Lastly, the basolateral projections of the posterior section are much more prominent in the two new species relative to that of *Euglossa crassipunctata* (it should be noted that these same differences hold for comparisons between the new species and *Euglossa sapphirina*). In addition, the lateral section of the gonostylus is significantly shorter and narrower in *Euglossa crassipunctata*, with a noticeably slender and elongate lateral lobe, while all of these structures are much broader and more prominent in *Euglossa moratoi* and *Euglossa clausi*. Undoubtedly, all of these species are closely related, but each is clearly distinct as evidenced by the male terminalia.

With the addition of the species described here, the *crassipunctata* species group comprises six species, which appear to fall into two subgroups. The first, the *crassipunctata* subgroup (*sensu strictissimo*) includes *Euglossa crassipunctata*, *Euglossa sapphirina*, *Euglossa moratoi*, and *Euglossa clausi* all with a triangular and well developed posterior mesotibial tuft. The second, or the *parvula* subgroup, consists of *Euglossa parvula* and *Euglossa pepei*, both with a posterior mesotibial tuft lacking or at most very small and inconspicuous (nearly vestigial). Both subgroups are represented in the Amazon and the Atlantic forests, but only the first subgroup is present in Central America.

In closing, it is significant to note the distinctiveness and apparent endemicity of *Euglossa pepei*, while *Euglossa moratoi* and *Euglossa clausi* are likely more common in collections, although undoubtedly misidentified as *Euglossa crassipunctata*. *Euglossa pepei* is presently known only from five specimens in a restricted area in Bahia, and the same region where species such as *Euglossa cyanochlora* Moure and *Exaerete salsai* Nemésio are also endemic. Among all species of the *crassipunctata* group, *Euglossa pepei* is the most distinctive in terms of both its external morphology and genitalia. It is greatly hoped that future collecting will bring more material of this species, particularly the unknown female, and permit a more thorough understanding of its biology and distribution.

## Supplementary Material

XML Treatment for Euglossa clausi

XML Treatment for Euglossa moratoi

XML Treatment for Euglossa pepei
